# A new online toolkit to support implementation research to enhance the use of digital innovations to End TB

**DOI:** 10.1371/journal.pdig.0000182

**Published:** 2023-02-02

**Authors:** Vanessa Veronese, Cecily Miller, Olumide Ogundahunsi, Saskia Den Boon, Yin Yin Xia, Dennis Falzon, Corinne S. Merle

**Affiliations:** 1 The Special Programme for Research and Training in Tropical Diseases (TDR), World Health Organization, Geneva, Switzerland; 2 Global Tuberculosis Programme, World Health Organization, Geneva, Switzerland; 3 The Central Office for Research and Development (CORD), University of Medical Sciences, Ondo City, Nigeria; 4 Chinese Centre for Disease Control, Beijing, China; Research Institute of the McGill University Health Centre, McGill University, CANADA

## Abstract

Digital technologies are playing an increasing role in the global response to tuberculosis (TB), however their effectiveness and impact are often shaped in the context in which they are implemented. Implementation research can help facilitate the effective introduction of digital health technologies in TB programmes. In 2020, the *Implementation Research for Digital Technologies and TB* online toolkit (IR4DTB) was developed and launched by the Special Programme for Research and Training in Tropical Diseases, and the Global TB Programme at the World Health Organization (WHO), to build local capacity for IR and promote the effective use of digital technologies within TB programmes. This paper describes the development and piloting of the IR4DTB toolkit, a self-learning tool designed for TB programme implementers. The toolkit comprises six modules reflecting key steps of the IR process, practical instructions and guidance on how to complete these steps, and real-word case studies to illustrate key learning points. This paper also describes the launch of the IR4DTB during a five-day training workshop with TB staff from China, Uzbekistan, Pakistan, Malaysia. The workshop included facilitated sessions on the IR4DTB modules, and provided an opportunity for participants to work with facilitators to develop a comprehensive IR proposal addressing an identified challenge related to the implementation and/or scale-up of digital health technologies for TB care in their home country. Post-workshop evaluation revealed high level of satisfaction among participants with the workshop content and format. The IR4DTB toolkit is a replicable model which can be used to strengthen the TB staff capacity to innovate within a culture of continuous collection of evidence. Through continued trainings and adaptation of the toolkit alongside the integration of digital technologies within TB prevention and care, this model has the potential to contribute directly to all components of the End TB Strategy.

## Author summary

Digital technologies are playing an increasingly important role in supporting national TB programmes (NTP) to further progress towards End TB goals. However, there is limited data on outcomes related to the implementation of such digital technologies for TB which restricts an understanding of how an intervention proven in one context may translate to another. Implementation research (IR) aims to understand the setting-specific barriers and facilitators that can shape the overall effectiveness and impact of a novel interventions in order to maximize their impact. In 2020, we developed an interactive online toolkit called the *Implementation Research for Digital Technologies and TB toolkit* to build capacity for IR among NTPs and other partners interesting in adopting digital tools to support their national TB responses. The toolkit was collaboratively developed and guides users through six modules which represent key steps in the IR process, resulting in the development of a comprehensive IR proposal. The toolkit was piloted during a workshop in China in 2020 with participants from four countries, who expressed satisfaction with the toolkit and workshop. The toolkit is publicly available from www.ir4dtb.org in English, French and Russian.

## Introduction

In the face of the ongoing tuberculosis (TB) epidemic, which in 2020 resulted in an estimated 10 million infections and 1.3 million deaths, predominately in low and middle income settings, innovative approaches to TB prevention and care have emerged as tools to enhance efforts in TB control [1]. The third pillar of the End TB strategy calls for both intensified research to optimize implementation and promote innovations, and the development and application of new interventions and tools as key strategies for pursuing global TB elimination targets [2]. Digital technologies are one example of such innovations with significant application potential to the TB response to improve patient outcomes and the pragmatic use of resources [3]. Digital technologies applied to TB have been classified into four core functions: patient care (e.g., video treatment support for TB patients), surveillance and monitoring (e.g., digital notification of TB cases), programme management (e.g., diagnostic device connectivity for TB) and eLearning (e.g., information resources platforms for patients on TB and smoking cessation) [2, 3]. Several digital health products that fit into one or more of these areas have been trialed for TB prevention and care for several years and are supported by the World Health Organization (WHO) [4, 5]. WHO has released evidence-based recommendations as well as implementation guidance on the use of supportive digital adherence technologies in TB programmes since 2017 [6, 7].

Much of the recent literature on digital technologies for TB has originated from high-income countries [8]. Evidence of impact of these technologies when applied to TB is varied and is often based on quantitative comparisons of treatment adherence and/or clinical outcomes [9]. Limited data on outcomes related to implementation of digital technologies for TB restricts an understanding of how an intervention proven in one context may translate to another, as challenges relating to implementation are often determined by local contexts and therefore uniquely specific. Moreover, implementation challenges may prevent interventions from achieving their full potential, leading to erroneous conclusions of ineffectiveness. For example, a recent randomized controlled trial (RCT) of a digital technology intervention for household TB contact investigation found no effect compared to the standard of care [10]. However, when evaluating the intervention using an implementation framework, key implementation challenges–such as low levels of trust and confidence in the digital tool among project staff, and hardware and software requirements that did not sufficiently consider the local context—that constrained the intervention and reduced its overall effectiveness were identified [11].

Implementation research (IR) is the systematic approach to recognizing, understanding and addressing health systems and implementation challenges, identifying optimal implementation options for a given setting and promoting the uptake of research into policy and practice [12]. IR represents an important interface between the availability of new tools and their use within a health system, making IR an important tool for facilitating the effective introduction of digital health interventions in TB programmes. Settings where the type or strength of evidence available in published literature differs, or where the overall impact of an intervention is likely to be influenced by the context in which it is implemented are examples of where IR can play an important role in guiding the introduction, use and scale up of new tools [12, 13]. Various factors determine which implementation research outcomes will be studied, including the identified implementation challenge, the stage of implementation of a new intervention, strategy or tool and also by the underlying framework that is being use, and commonly include outcomes such as acceptability, feasibility, cost effectiveness, adoption and effectiveness [12–14]

In order to build local capacity for IR and promote the effective use of digital technologies within TB programmes, The Special Programme for Research and Training in Tropical Diseases (TDR) and the Global TB Programme (GTB) at WHO developed the *Implementation Research for Digital Technologies and TB* online toolkit (IR4DTB) to evaluate the implementation and scale up of digital innovations across the TB continuum of care [15]. This paper describes the development, features and roll out of the IR4DTB tool.

## Methods

### Conceptualization of the IR4DTB toolkit

In December 2018, GTB and the European Respiratory Society (ERS) convened a first technical consultation on digital innovations, TB and implementation research. This meeting brought together donors, technical experts, researchers, non-governmental organizations and implementers from public and private sectors to discuss experiences and perspectives in implementation science methods to help scale up digital innovations under programmatic conditions to support efforts against TB. It was during this meeting that the idea for a practical, online toolkit first emerged as a strategy to address the demand from national TB programmes (NTPs) and other implementers to mount IR projects specifically geared towards evaluating digital technologies for TB prevention and care under routine programmatic conditions. Work on the toolkit then commenced in late 2019 with support from an external consultant hired by TDR to lead the development of the toolkit.

### Objectives of the IR4DTB toolkit

The authors conceived and built the toolkit with the aim of providing a self-learning tool for TB programme implementers to plan and conduct IR. The expected objectives of the toolkit are: 1) increase capacity among NTP staff and other TB programme implementers to design and conduct IR focused on digital technologies and; 2) facilitate the development of comprehensive IR proposals that could be used to seek fundraising from potential donors.

The IR4DTB toolkit was developed by TDR and WHO using an iterative process and in consultation with various NTP staff from countries with high TB burdens who took part in the 2018 meeting (South Africa, China, Russian Federation, India and Brazil). The IR4DTB was developed as an adaptation of the Implementation Research toolkit first developed by TDR and partners in 2014, which was developed as a self-learning tool to help researchers and programme implementers systematically identify barriers to effective implementation of health programmes, strategies and interventions and to support the planning and conduct of research aimed at understanding and addressing such bottlenecks [12]. This toolkit was developed under the guidance of steering committee comprised of external experts in implementation research, with contributions and input from over 200 researchers, academics, disease control programme managers, policy- makers, health administrators, communication experts and journalists who also provided feedback on its use [12]. Consequently, this toolkit was used as a validated resource which underpinned the subsequent IR4DTB toolkit. Peer review was also an integral part of the development and validation of the IR4DTB, with regular revisions of progress and content conducted by staff from GTB and TDR throughout the process, as well as feedback sought from external experts on draft iterations of the toolkit. In February 2020, a two-day technical meeting was held at the WHO Headquarters in Geneva, Switzerland, convened by WHO and the ERS on digital innovations, TB and implementation research. This meeting brought together funders, partners, representatives of NTP, civil society and scientists to discuss the challenges and opportunities when evaluating, integrating and scaling up digital technologies under programmatic settings. During this meeting, a dedicated session was held to discuss and validate the development of the toolkit [16]. The finalized toolkit was piloted during a five-day hybrid workshop (held in person in Beijing, China with virtual participation; described further below), at the end of which a questionnaire was self-administered among participants to evaluate their perception of IR4DTB and provide an opportunity to collect feedback for improvement. The questionnaire collected basic socio-demographic data from participants (current occupation; number of years of research experience; self-rated level of professional experience with any digital technologies: none/limited/moderate or sound knowledge/extensive experience or expert level user), included a range of Likert-scale questions to assess perceptions related to the usefulness of the toolkit and training, and ended with a series of open-ended questions about the strengths and weakness of the IR4TBD site and suggestions for future changes or improvements. Quantitative data from the survey was analyzed using Microsoft Excel.

## Results

The finalized IR4DTB toolkit is organized around six modules reflecting key steps of the IR process (Table 1). These steps were adapted for the express purposes of IR projects focused on any of the four digital functions of patient care, programme management, eLearning and surveillance and monitoring [2]. Each module provides practical instruction and guidance for users around the key IR steps and has been designed to be followed in a step-wise fashion.

**Table 1:** Overview of the six modules of IR4DTB, based on the key steps in the conduct of IR on digital technologies for TB.

Module	Module objectives	Sub-module headings
*Preparing for IR*	Provide an overview of key IR concepts Guide users through the steps to identify an implementation challenge that can be further explored using an IR approach Understand the components of a typical IR proposal structure	Setting the scene for IR Engaging relevant stakeholders Understanding the implementation challenge Reviewing the literature uilding an IR team Developing an IR proposal
*Developing IR objectives and questions*	Understand how to turn an identified implementation challenge into an IR objective Learn how to develop an answerable research question Learn how to prepare a proposal introduction	Implementation research objectives and questions Writing an IR proposal introduction
*Research methods*	Understand what a research outcome is and how to measure it Learn how to select an appropriate study design Understand how to identify and select research participants Understand key ethical principles and considerations in IR Understand the process of applying for and receiving ethical clearance and learn how to develop informed consent forms	Implementation outcomes Research instruments and study populations Study design Methodology Economic evaluations IR ethics
*Data management and analysis*	Understand requirements for data management and strategies for ensuring data quality Understand key data analysis methods for qualitative and quantitative data Develop a data analysis plan	Data collection Data quality and management Data analysis
*Planning and conducting IR*	Understand the requirements and planning needed to launch an IR study Develop a project plan to guide study implementation Develop a monitoring plan	Planning for an IR study Monitoring the implementation of an IR study Launching and conducting an IR study
*Knowledge translation*	Understand the importance of knowledge translation for IR Develop a strategy on promoting translation and uptake of IR findings by stakeholders and health systems	Uptake of IR findings Developing a dissemination plan Dissemination strategies

To support the development of a comprehensive IR proposal, a generic proposal template is included, and the toolkit’s practical activities have been designed to feed into different components of the IR proposal. Real life case studies taken from country experiences conducting IR on digital technologies for TB are included throughout the toolkit to illustrate how various IR concepts described in the toolkit can be applied to programmatic realities and are designed to give a practical example of how key learning outcomes of the toolkit can be practically applied. In total, five case studies have been included which cover each of the four digital functions (Table 2).

**Table 2:** Overview of the case studies across the four digital functions in the IR4DTB.

Digital function	Case study title
Patient care	*Electronic medication monitors in China* *Mobile cash transfers as enablers for TB patient support in India*
Surveillance and monitoring	*National electronic TB recording and reporting system in South Africa*
Programmatic management	*Connected TB diagnostics platform in South Africa*
eLearning	*eLearning platform for HIV-associated TB in Brazil*

### Delivery of the first training workshop based on the IR4DTB toolkit

The toolkit was officially launched during a five-day training workshop in November 2020, co-organised with the Chinese Anti-TB Association (CATA) and China Centre for Disease Control (CCDC). The workshop was attended by staff from NTPs and Ministry of Health (MoH) in China, Uzbekistan, Malaysia, and Pakistan. In light of COVID 19-related travel restrictions and safety measures, the workshop adopted a hybrid approach involving in-person attendance for Chinese participants (in Beijing) and virtual participation for participants and session coordinators based outside of China using Zoom. Facilitation was provided by in-country personnel and remotely by GTB and TDR teams.

Prior to the workshop, attendees were offered the opportunity to participate in an IR massive online open course (MOOC) facilitated by TDR which provided a baseline level of IR knowledge to be further expanded upon during the workshop [17]. At the end of the MOOC, participants were asked to develop a brief outline of a potential IR study which was used as the foundation for the comprehensive study proposal developed by research groups assembled for the workshop.

The training workshop provided sessions on how to conduct IR when novel digital technologies are introduced or scaled up using IR4DTB as the core teaching tool. The course included a range of sessions addressing topics from each of the six IR4DTB modules and delivered as virtual, didactic, short seminars by staff from GTB, TDR or invited external experts. Following the sessions, participants were given the opportunity to work in small research groups to apply the new knowledge and develop a comprehensive study proposal which addressed an identified challenges related to the effective implementation and/or scale-up of digital health technologies for TB care in their home countries. Each group was paired with an international facilitator who provided virtual technical support to groups in the development of their study proposals, which were presented to the wider group on the study groups presented their IR proposal to the group for peer-review.

The workshop was attended by 27 participants from three countries; four from Pakistan, six from Malaysia and 17 from China (the team from Uzbekistan did not complete the workshop due to difficulties with connectivity). Based on responses collected by a post-workshop survey (completed by 16 of 27 participants; 59%), most participants identified as clinicians or other medical staff, and reported less than two years of research experience and limited experience using digital technologies (Table 3).

**Table 3:** Workshop participant profiles (N = 16).

	n (%)
**Current occupation**	
Clinician or other medical staff	9 (56.3)
Researcher	3 (18.8)
NTP staff	2 (12.5)
Other	2 (12.5)
**Research experience (years)**	
None	1 (6.3)
<2	5 (31.3)
2 to 5	3 (18.8)
5 to 10	3 (18.8)
10>	4 (25)
**Self-rated level of professional experience with any digital technologies**	
None	4 (25)
Limited experience	8 (50)
Moderate experience/ sound knowledge	3 (18.9)
Extensive experience/ expert level	1 (6.3)

The workshop resulted in the development of four relevant study proposals (Table 4). At the time of writing (October 2022), all four teams had finalized their full study protocols, and three from China received internal funding and were completed, while Pakistan’s study proposal was recently included as part of a funding grant application to a TB-focused multi-lateral organization. Regular calls were held approximately every two months since the workshop between study teams and staff at GTB and TDR to share updates and provide ad hoc technical support. Manuscripts have been submitted for two of the three studies from China and are currently under review, while the third manuscript is currently being prepared for submission (Table 4).

**Table 4:** IR studies developed during IR4DTB training workshop.

Country	IR study title	Study update (as of October 2022)
China	Evaluating the acceptability of computer-assisted detection (CAD) programmes by health care providers for evaluation for TB in primary care settings	Study completed, manuscript currently being developed
China	Introducing and evaluating a new digital Management System (e-TBMS) in Hubei, China	Study completed, manuscript submitted for publication (“*Evaluating the impact of a new digital TB patient management system on follow up and treatment outcomes in Wuhan*, *China*”)
China	Scaling up different types of electronic medication monitors to assist TB medication management in China	Study completed, manuscript submitted for publication ("Current status and future prospects of TB digital health tools use in China: A national cross-sectional study")
Pakistan	Improving TB case detection through contact tracing using digital technology in Pakistan	Study protocol developed, awaiting confirmation of funding before starting study

The post-workshop survey sought to evaluate participants’ satisfaction and perceptions of the workshop through a range of statements about the usefulness, format, content and benefit of the IR4DTB tool and training. Responses were provided using a five-point Likert scale (*strongly agree*, *agree*, *neutral*, *disagree*, *strongly disagree*). The survey also included four short-answer questions to elaborate on the strengths and weakness of the tool and training and to solicit feedback about suggestions for improvement or changes.

Responses to Likert-scale questions revealed that most participants found that both the training and the IR4DTB tool were useful in imparting knowledge and skills on how to conduct IR for digital technology for TB care (Table 5).

**Table 5:** Participant agreement with statements pertaining to usefulness of IR4DTB tool and workshop (n = 27).

	Strongly agree n (%)	Agree n (%)	Neutral n (%)	Disagree n (%)	Strongly disagree n (%)
*IR4DTB workshop*					
*The content of this training was well aligned to the stated objectives and purpose of the workshop*	100	0	0	0	0
*The organization of the workshop (e*.*g*. *logistics*, *connectivity) achieved the course objectives*	14 (87.5%)	2 (12.5%)	0	0	0
*The format and method of delivery used in this workshop and knowledge training allowed me to effectively learn and master new knowledge and/or skills*	11 (68.8%)	5 (31.3%)	0	0	0
*The training helped me to understand about both implementation research and the role of digital health for TB*	12 (75%)	3 (18.8%)	1 (6.3%)	0	0
*I was satisfied with the choice of presenters for this course*	14 (87.5%)	2 (12.5%)	0	0	0
*IR4DTB toolkit*					
*The IR4DTB is a useful resource in guiding me through the development of a IR study proposal*	14 (87.5%)	2 (12.5%)	0	0	0
*I found the IR4DTB easy to navigate / use*	11 (68.7%)	4 (25.0%)	0	1 (6.3%)	0
*Without the IR4DTB*, *I would not have developed my IR study proposal*	7 (43.8%)	4 (25.0%)	4 (25.0%)	1 (6.3%)	0

The final four questions of the survey allowed for short answer responses to questions about the strengths and weakness of the IR4TBD site and suggestions for future changes or improvements. The specific and comprehensive guidance on how to develop an IR proposal was commonly highlighted as a strength. Other strengths included the “professional and detailed website content”, and the “clear and stepwise approach” to developing an IR proposal. Suggestions for improvement largely focused on the need for translation of the toolkit content into other languages and the inclusion of audio-visual material. Some participants recommended face-to-face delivery for future workshops and one participant felt that more time should be given to sufficiently cover the module on research methods and data analysis. In response to this feedback, work is currently underway to translate the IR4DTB into French to extend the availability of this tool to Francophone African settings. Funding is also being sought to enable to development of a Russian-language IR4DTB toolkit. User evaluation on the toolkit and training will be sought during future workshops to enable ongoing opportunity for feedback. In addition, a function has been added to the IR4DTB online site that allows users to submit comments and/or questions at any time.

## Discussion

The virtual nature of the IR4DTB tool and associated training workshop has allowed us to expand the reach and conduct training despite COVID-19 related restrictions on movement and gatherings. Additional benefits include cost savings and the ability to reach a greater number of people at one time. Nonetheless, the desire to have in-person training in future is fully understood and opportunities will be sought to create blended meetings as was done in our workshop in China. Continuing to expand the availability of the IR4DTB toolkit beyond English-speaking settings will be an ongoing priority. Discussions are ongoing with WHO regional offices to replicate the workshop for African and Russian-speaking countries. Moreover, in response to user demand, a “workbook” presenting the practical website components in a downloadable format is being elaborated.

Digital tools have demonstrated significant potential in contributing to COVID-19 control measures such as, symptom screening and monitoring, contact tracing, quarantine and self-isolation and clinical management [18, 19]. The potential of technological and broader contextual factors to constrain the overall efficiency of these rapidly deployed tools has been noted [19], underscoring the importance of IR to ensure that the benefit of these new tools may be fully realized. In this paper we describe how our IR toolkit—conceived before COVID-19 emerged and developed during the pandemic months, could help to investigate such barriers and come up with fully fledged study proposals.

There are several limitations that must be considered in the context of this tool and the evaluation results. First, social desirability bias may have influenced some of the responses and ratings provided by workshop participants and users of IR4DTB. Second, given the small number of respondents, it is not possible to make generalizations about the broader utility of IR4DTB, particularly among those who may use IR4DTB as a self-directed study tool without the workshop and additional instruction. The “workbook” in development is intended to facilitate self-paced learning. Last, while IR4DTB has an important role to play in building IR capacity and providing guidance on the development of IR studies, study teams who go on to conduct IR studies will still require local expertise for fine tuning the project–beyond what can be provided by technical support from WHO staff–and will require a sufficient level of national willingness and interest in investing in digital technologies for improving TB care as a precursor to success.

In conclusion, we report the creation and initial use of an implementation research toolkit as a replicable model to strengthen the capacity of staff working in TB care to innovate within a culture of continuous collection of evidence. Through continued trainings and adaptation of the toolkit alongside the integration of digital technologies at key stages of the continuum of TB prevention and care, this model has the potential to contribute directly to all components of the End TB Strategy at this critical juncture.

## References

[1] WHO. Global TB Report 2021. Geneva: WHO, 2022

[2] WHO. The End TB strategy: global strategy and targets for tuberculosis prevention, care and control after 2015. Geneva: WHO; 2015.

[3] Falzon D, Timimi H, Kurosinski P, Migliori GB, Van Gemert W, Denkinger C, et al. Digital health for the End TB Strategy: developing priority products and making them work. Eur Respir J. 2016;48(1):29–45. doi: 10.1183/13993003.00424-2016 27230443 PMC4929075

[4] Alipanah N, Jarlsberg L, Miller C, Linh NN, Falzon D, Jaramillo E, et al. Adherence interventions and outcomes of tuberculosis treatment: A systematic review and meta-analysis of trials and observational studies. PLoS Med. 2018;15(7):e1002595. doi: 10.1371/journal.pmed.1002595 29969463 PMC6029765

[5] World Health Organization. Electronic recording and reporting for tuberculosis care and control. Geneva: World Health Organization; 2012. Contract No.: WHO/HTM/TB/2011.22.

[6] World Health Organization. Guidelines for treatment of drug-susceptible tuberculosis and patient care: 2017 update. Geneva: World Health Organization,; 2017 2017. Contract No.: WHO/HTM/TB/2017.05.

[7] World Health Organization. Handbook for the use of digital technologies to support tuberculosis medication adherence. Geneva: World Health Organization; 2017. Contract No.: WHO/HTM/TB/2017.30.

[8] Lee Y, Raviglione MC, Flahault A. Use of Digital Technology to Enhance Tuberculosis Control: Scoping Review. J Med Internet Res. 2020;22(2):e15727. 32053111 10.2196/15727 PMC7055857

[9] Ngwatu BK, Nsengiyumva NP, Oxlade O, Mappin-Kasirer B, Nguyen NL, Jaramillo E, et al. The impact of digital health technologies on tuberculosis treatment: a systematic review. Eur Respir J. 2018;51(1). doi: 10.1183/13993003.01596-2017 29326332 PMC5764088

[10] Davis JL, Turimumahoro P, Meyer AJ, Ayakaka I, Ochom E, Ggita J, et al. Home-based tuberculosis contact investigation in Uganda: a household randomised trial. ERJ Open Res. 2019;5(3). doi: 10.1183/23120541.00112-2019 31367636 PMC6661318

[11] Meyer AJ, Armstrong-Hough M, Babirye D, Mark D, Turimumahoro P, Ayakaka I, et al. Implementing mHealth Interventions in a Resource-Constrained Setting: Case Study From Uganda. JMIR Mhealth Uhealth. 2020;8(7):e19552. doi: 10.2196/19552 32673262 PMC7385635

[12] TDR. TDR Implementation Research Toolkit 2014 [https://adphealth.org/irtoolkit/.

[13] Peters D H, Adam T, Alonge O, Agyepong IA, Tran N. Implementation research: what it is and how to do it BMJ 2013; 347: f6753 doi: 10.1136/bmj.f6753 24259324

[14] Squire SB, Ramsay AR, van den Hof S, Millington KA, Langley I, Bello G, et al. Making innovations accessible to the poor through implementation research. Int J Tuberc Lung Dis. 2011;15(7):862–70. doi: 10.5588/ijtld.11.0161 21682960

[15] WHO. Implementation Research for Digital Technologies and TB: a toolkit for evaluating the implementation and scale up of digital innovations across the TB continuum of care. 2020 [https://ir4dtb.org/.

[16] WHO. Digital Innovations, TB and implementation research: report of a joint meeting organised by the World Health Organization and the European Respiratory Society. Geneva: WHO [https://www.who.int/docs/default-source/documents/tuberculosis/digital-innovations-tb-and-implementation-research.pdf?sfvrsn=631377c7_1].

[17] TDR. Massive open online course (MOOC) on implementation research: infectious diseases of poverty 2021 [https://tdr.who.int/home/our-work/strengthening-research-capacity/massive-open-online-course-(mooc)-on-implementation-research.

[18] Whitelaw S, Mamas MA, Topol E, Van Spall HGC. Applications of digital technology in COVID-19 pandemic planning and response. Lancet Digit Health. 2020;2(8):e435–e40. doi: 10.1016/S2589-7500(20)30142-4 32835201 PMC7324092

[19] Gasser U, Ienca M, Scheibner J, Sleigh J, Vayena E. Digital tools against COVID-19: taxonomy, ethical challenges, and navigation aid. Lancet Digit Health. 2020;2(8):e425–e34. doi: 10.1016/S2589-7500(20)30137-0 32835200 PMC7324107

